# Looks Can Be Deceiving: Diagnostic Power of Exome Sequencing in Debunking 15q11.2 Copy Number Variations

**DOI:** 10.3390/genes15111441

**Published:** 2024-11-07

**Authors:** Camilla Meossi, Alessia Carrer, Claudia Ciaccio, Laura Pezzoli, Lidia Pezzani, Rosa Maria Silipigni, Francesca L. Sciacca, Romano Tenconi, Silvia Esposito, Arianna De Laurentiis, Chiara Pantaleoni, Paola Marchisio, Federica Natacci, Stefano D’Arrigo, Maria Iascone, Donatella Milani

**Affiliations:** 1 Fondazione IRCCS Ca’ Granda Ospedale Maggiore Policlinico, 20100 Milan, Italy; lidia.pezzani@policlinico.mi.it (L.P.); rosamaria.silipigni@policlinico.mi.it (R.M.S.); paola.marchisio@policlinico.mi.it (P.M.); federica.natacci@policlinico.mi.it (F.N.); 2 Department of Health Sciences, University of Milan, 20100 Milan, Italy; alessia.carrer@unimi.it; 3 Fondazione IRCCS Istituto Neurologico C. Besta, 20100 Milan, Italy; claudia.ciaccio@istituto-besta.it (C.C.); f.sciacca@istituto-besta.it (F.L.S.); silvia.esposito@istituto-besta.it (S.E.); arianna.delaurentiis@istituto-besta.it (A.D.L.); chiara.pantaleoni@istituto-besta.it (C.P.); stefano.darrigo@istituto-besta.it (S.D.); 4 Laboratory of Medical Genetics, ASST Papa Giovanni XXIII, 24100 Bergamo, Italy; lpezzoli@asst-pg23.it (L.P.); miascone@asst-pg23.it (M.I.); 5 Pediatric Unit, ASST Papa Giovanni XXIII, 24100 Bergamo, Italy; 6 Clinical Genetics Unit, Department of Women and Children’s Health, University of Padova, 35100 Padova, Italy; romano.tenconi@unipd.it

**Keywords:** 15q11.2 microdeletion, 15q11.2 microduplication, BP1-BP2 CNVs, exome sequencing, alternative diagnosis, double diagnosis, rare diseases

## Abstract

**Background/Objectives**: The pathogenetic role of 15q11.2 Copy Number Variations (CNVs) remains contentious in the scientific community, as microdeletions and microduplications in this region are linked to neurodevelopmental disorders with variable expressivity. This study aims to explore the diagnostic utility of Exome Sequencing (ES) in a cohort of pediatric patients with 15q11.2 CNVs. **Methods**: We enrolled 35 probands with 15q11.2 microdeletions or microduplications from two genetic centers between January 2021 and January 2023. Chromosomal Microarray Analysis (CMA) and ES were performed with written consent obtained from all parents. Pathogenic variants were classified according to ACMG guidelines. **Results**: CMA identified additional pathogenic CNVs in 3 of 35 children (9%). Subsequent ES revealed likely pathogenic or pathogenic variants in 11 of 32 children (34%). Notably, a higher percentage of isolated autism spectrum disorder (ASD) diagnoses was observed in patients without other CNVs or point mutations (*p* = 0.019). **Conclusions**: The ES analysis provided a diagnostic yield of 34% in this pediatric cohort with 15q11.2 CNVs. While the study does not dismiss the contribution of the CNV to the clinical phenotype, the findings suggest that ES may uncover the underlying causes of neurodevelopmental disorders. Continuous monitoring and further genetic testing are recommended for all 15q11.2 CNV carriers to optimize clinical management and familial counseling.

## 1. Introduction

The 15q11.2 genomic region contains five clusters of Low Copy Repeats that predispose to several Copy Number Variations (CVNs) by misalignment during meiosis [1].

In particular, proximal breakpoints BP1 and BP2 flank a recurrent CNV approximately 500 kb long that contains four evolutionary highly conserved genes (*NIPA1*, *NIPA2*, *CYFIP1*, *TUBGCP5*) [2], which have been reported to be critical for neurodevelopment and function [3].

Both 15q11.2 microdeletion and microduplication have been associated with neurodevelopmental disorders, neuropsychiatric/behavioral disturbances, and other mild clinical features [4,5,6].

The pathogenetic role remains debated within the scientific community, as the CNV exhibits incomplete penetrance and variable expressivity. Actually, most carriers present with mild or no symptoms, and pathological features affect only 10% of them [7].

Clarifying this dilemma is important to reduce the risk of inaccurate diagnoses that could have significant implications for both prenatal counseling and follow-up care. We previously clinically described a pediatric cohort of BP1–BP2 CNV carriers affected by neurodevelopmental disorders [8].

In this study, we supported the hypothesis that the high frequency of neurodevelopmental disorders in some cohort of 15q11.2 microdeletion/microduplication carriers could be due to a sampling bias: Chromosomal Microarray Analysis (CMA) has long been the first-tier diagnostic test for individuals with developmental delay or congenital anomalies, leaving unexplored possible further point mutations in other genomic regions.

Therefore, we searched for additional genetic variants that may contribute to phenotype in order to find an explanation for the wide inter- and intrafamilial variability of the debated 15q11.2 CNV.

## 2. Materials and Methods

From January 2021 to January 2023, a total of 35 probands and their biological parents were enrolled in two reference centers for genetic evaluation of children affected by intellectual disability and other neurodevelopmental disorders.

The only inclusion criterion was the presence of 15q11.2 BP1-BP2 microdeletion or microduplication. Probands were instead excluded if biological parents were not available for testing. ES was not performed if CMA highlighted the presence of additional genomic rearrangements.

Besides anamnestic data collection, all participants underwent physical examination and neuropsychiatric assessment. Data were collected by medical record review regarding the following points: sex, age, course of pregnancy and delivery, developmental delay, autism spectrum disorders, and other behavioral problems, growth pattern, dysmorphic features, vision and hearing problems, and other systems’ involvement, including cardiac, musculoskeletal, gastrointestinal, and genitourinary abnormalities.

International standardized scales have been used to assess the development (Griffiths and WISC-IV) and possible autism spectrum disorders. A neuropsychiatric diagnosis was made according to the Diagnostic and Statistical Manual of Mental Disorders (DSM V) criteria.

Comprehensive pre-test counseling was undertaken for all the patients by clinical geneticists.

Molecular karyotyping was performed through array-CGH using SurePrint G3 Human CGH Array Kit, 8 × 60 K and 4 × 180 K (Agilent Technologies, Santa Clara, CA, USA). Labeling and hybridization were performed according to the manufacturer’s protocol. Agilent Feature Extraction was used to quantify the fluorescence of the scanned images and Cytogenomics 2.7 software was used for data analysis. A CNV call was performed using the ADAM-2 algorithm. Probe positions are referred to as hg19/GRCh37.

Trio-ES has been performed at the Medical Genetics Laboratory of ASST Papa Giovanni XXIII of Bergamo. Written informed consent and the trio-ES analysis were obtained from the parents of the patients.

Genomic DNA was extracted from peripheral blood samples of probands and parents using standard procedures. The exonic regions and flanking splice junctions of the genome were captured using the Clinical Research Exome v.2 kit (Agilent Technologies, Santa Clara, CA, USA). Sequencing was performed on a NextSeq500 Illumina system with 150 bp paired-end reads. Reads were aligned to human genome build GRCh37/UCSC hg19 and analyzed for sequence variants using a custom-developed analysis tool, already validated and standardized [9]. The variant call file, including single nucleotide polymorphism and indels, was annotated by querying population frequencies databases and mutation databases, including the Genome Aggregation Database (http://gnomad.broadinstitute.org/ accessed on 15 January 2021), ClinVar (https://www.ncbi.nlm.nih.gov/clinvar/ on 15 January 2021) and Human Gene Mutation Database Professional (HGMD, Release 2017.4). Variants were classified based on ACMG guidelines [10]. The potential causative variants were subsequently confirmed by Sanger sequencing in the proband and parents using an independent DNA sample.

The statistical analysis was evaluated using Statistical Package SPSS type 2 for social science software. We calculated the frequencies (number and percentile) for the qualitative variables and the median, the first (Q1) and the third (Q3) quartiles for the quantitative ones. We used the Chi-squared test and Fisher’s exact test. A *p*-value < 0.05 was considered statistically significant.

## 3. Results

### 3.1. Clinical Description

A total of 21 out of 35 recruited patients were males (60%) and 14 were females (40%). The average age was 11 years and 3 months. The 15q11.2 CNV was inherited from a healthy parent in 86% of patients (Supplementary Table S1). Our analysis did not reveal a significant correlation between the size of the 15q11.2 CNV and the severity of the patients’ symptoms.

The course of pregnancy was regular in all cases. Most patients showed a normal growth. Weight and height were below the 3rd centile in only two patients and above 97° percentile in one patient. OFC was harmonious compared to the other auxological parameters in most cases. Among patients only three showed an absolute microcephaly, three absolute macrocephaly and one relative macrocephaly.

As for the other clinical features, 10 patients presented with malformations, mostly congenital heart disease (Supplementary Table S1).

**Figure 1:**
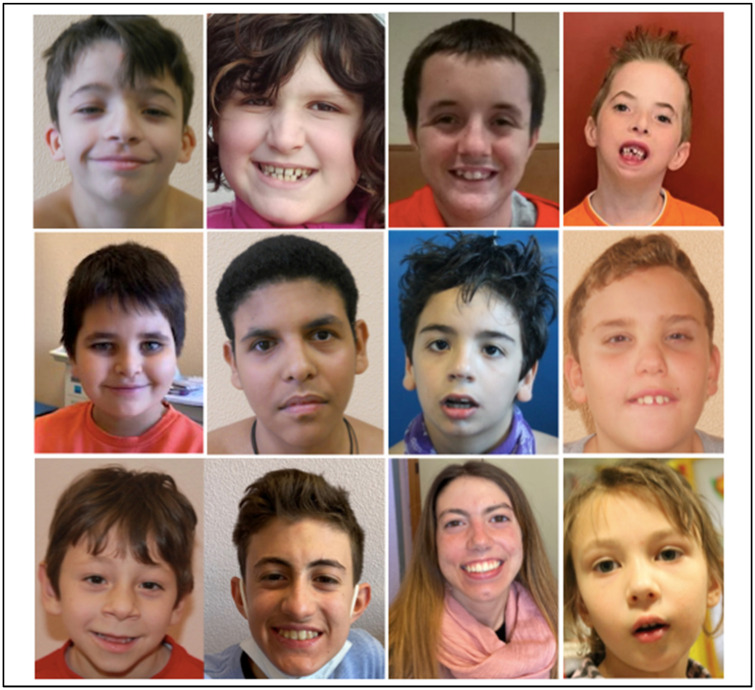
Face pictures of some 15q11.2 CNV carriers with dysmorphic features. Neither the subgroup of 15q11.2 microdeletions nor the microduplication presented with recurrent features. Top row from left to right: Patient 4, Patient 25, Patient 8, Patient 7; middle row from left to right: Patient 20, Patient 23, Patient 15, Patient 17; bottom row from left to right: Patient 9, Patient 6, Patient 5, Patient 13.

Facial dysmorphisms were found in about 40% of cases; neither the subgroup of 15q11.2 microdeletions nor the microduplication presented with recurrent features (Figure 1).

All children presented with neurodevelopmental disorders. Neurodevelopmental delay or intellectual disability was the most reported feature (91% of patients), followed by autism spectrum disorders (23%).

EEG showed anomalies in 23% of cases. MRI was performed in seven patients with 15q11.2 microdeletion and fifteen with 15q11.2 microduplication with abnormal results in 14 cases. (Supplementary Table S1).

All subjects had performed CMA as a first-tier genetic test. A total of 32 patients carried only the 15q11.2 BP1-BP2 CNV, but further likely pathogenetic or pathogenetic CNVs had been highlighted in three children, in particular: Patient 2 arr[hg19]1q42.2q43(232,746,308 − 242,987,796) × 1; the CNV is not described in the literature and not reported in gnomAD, DGV, or DECIPHER. Patient 3 arr[hg19]16p13.11(15,492,317 − 16,276,115) × 3; the recurrent CNV is associated with the “16p13.11 microduplication syndrome”. Patient 10: arr[hg19]15q11.2q12(23,597,805 − 27,813,937) × 3; the CNV is well-known and associated with "chromosome 15q11-q13 duplication syndrome” (MIM# 608636) (Supplementary Table S1). These three patients did not perform trio-ES analysis.

### 3.2. Trio-ES Analysis

Trio-ES analysis revealed likely pathogenetic variants or pathogenetic variants in 11 out of 32 patients (23%) and variants with uncertain significance in four other patients (13%). ES results are listed in Table 1.

Excluding from the sample the cases with other pathogenetic CNV at CMA, the detection rate of the ES analysis in our cohort was 47% (variant of uncertain significance, VOUS 13%; likely pathogenic variant, LP/pathogenic variant, P 34%).

**Figure 2:**
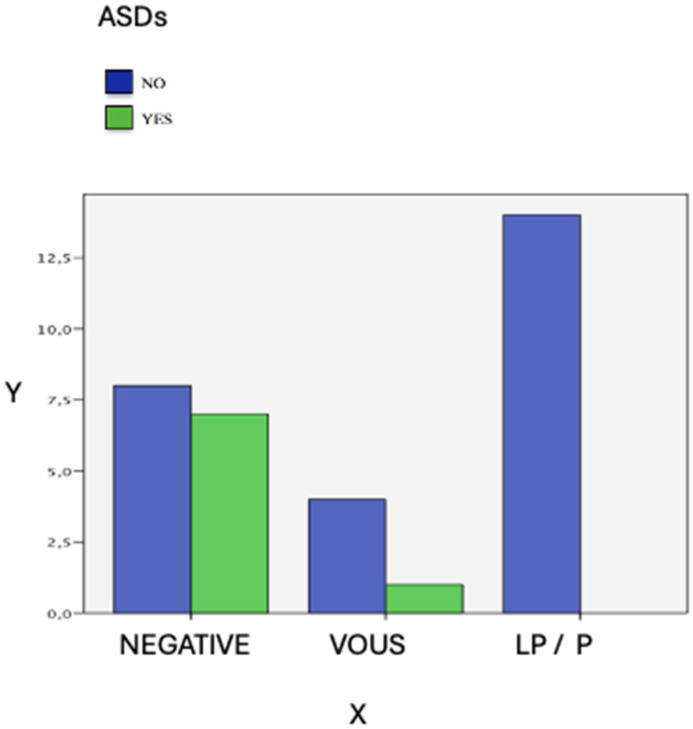
Trio-ES detection rate in isolated Autism Spectrum Disorder. Among 15q11.2 CNV carriers affected by isolated ASDs, the results of ES are displayed on the *x*-axis, while the number of patients is shown on the *y*-axis. ES did not identify any Likely Pathogenic or Pathogenic variants in any subjects; a Variant of Uncertain Significance was reported in only one patient. Points in Blue indicate patients without a diagnosis of ASDs, while points in Green indicate patients with a diagnosis of ASDs (see legend for details).

A noteworthy statistically significant finding is the higher percentage of isolated ASD diagnoses in patients who do not have other CNVs in CMA or variant points in WES analysis (Pearson square chi: p=0.019). (Figure 2).

**Figure 3:**
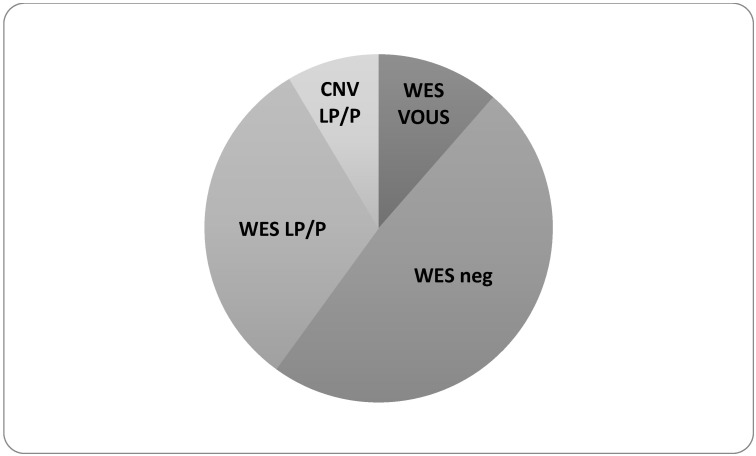
Diagnostic yield of Array CGH and trio-ES in 15q11.2 CNV carriers. In three out of 35 (9%) children CMA detected additional pathogenic or likely pathogenic genomic arrangements (CNV LP/P). ES showed a very high detection rate (31%) of pathogenic or likely pathogenic variants (WES LP/P). In further 4 patients (11%), a variant of uncertain significance has been reported (WES VOUS). In 17 patients (49%), the ES analysis was negative (WES neg).

Overall, our cohort of 15q11.2 carriers revealed the following genetic variants (Figure 3):

- Three patients (9%) with additional pathogenic CNV.
- Eleven patients (31%) with likely pathogenetic or pathogenetic variants highlighted in ES analysis.
- Four patients (11%) with VOUS highlighted in ES analysis.
- Seventeen patients (49%) negative for ES analysis.

**Table 1:** Trio-ES results. The table shows trio-ES results in out cohort of 15q11.2 CNV carriers. Mat: maternal; Pat: paternal; P: pathogenic variant; LP: likely pathogenic variant; VOUS: variant of uncertain significance.

Case	Gene	Variant	Inheritance	ACMG Classification	ACMG Evidence Support
Patient 5	*ZMYM2*	c.[1546C>T]; p.[Arg516ter]	de novo	*LP*	*PM2*, *PVS1*, *PS2*
Patient 6	*CNKSR2*	c.[741+4A>G]	mat	*VOUS*	*PM2*, *PP3*
Patient 7	*PSMD12*	c.[1246del]; p.[Gln416fs]	de novo	*LP*	*PM2*, *PVS1*, *PS2*
Patient 9	*CTNNB1*	c.[1304dup]; p.[Met436AspfsTer21]	de novo	*LP*	*PM2*, *PVS1*, *PS2*
Patient 12	*CACNA1I*	c.[3620C>T]; p.[Ser1207Phe]	de novo	*VOUS*	*PM2*, *PP2*, *PP3*, *PS2*
Patient 13	*GRIN2B*	c.[39_40del]; p.[Trp13fs]	de novo	*LP*	*PM2*, *PVS1*, *PS2*
Patient 15	*PURA*	c.[399_400dup]; p.[Gln134fs]	de novo	*LP*	*PM2*, *PVS1*, *PS2*
Patient 17	*OPHN1*	c.[595C>G;597+3A>C]	mat	*LP*; *LP*	*PM2*, *PP3*, *PS3*
Patient 22	*CLCN4*	c.[847A>G]; p.[Ser283Gly]	de novo	*LP*	*PM2*, *PP3*, *PS2*
Patient 24	*SOS1*	c.[671A>C]; p.[Lys224Thr]	mat	*VOUS*	*PM2*
Patient 25	*GRIN2A*	c.[2168+1G>A]	de novo	*LP*	*PM2*, *PVS1*, *PS2*
Patient 27	*MECP2*	c.[419C>T]; p.[Ala140Val]	de novo	*P*	*PM2*, *PM1*, *PP5*, *PS2*
Patient 29	*NAA15*	c.[1492dup]; p.[Met498fs]	de novo	*LP*	*PM2*, *PVS1*, *PS2*
Patient 30	*GEMIN5*	c.[2200C>T]; p.[Leu734Phe]; c.[3232G>A]; [Val1078Ile]	mat;pat	*VOUS*; *VOUS*	*PM2*; *PM2*
Patient 34	*VARS1*	c.[1630G>C]; p.[Asp544His]; c.[2348A>G]; [Asp783Gly]	mat;pat	*LP*; *LP*	*PM2*; *PM2*

## 4. Discussion

At the state-of-the-art, the 15q11.2 CNV is considered a likely pathogenic genomic rearrangement characterized by incomplete penetrance and variable expressivity [5]. The microdeletion was first described in 2007 in a child affected by hypotonia, speech impairment, and neurological disorders, but also in his father, who presented with similar but milder clinical features [11]. Subsequent cohort studies have associated the CNV with developmental delay/intellectual disability, learning difficulties, autism spectrum disorders, psychiatric and behavioral disturbances, seizures, brain anomalies, and congenital malformations [6,12,13,14,15,16,17,18]. Dysmorphic traits were described in more than 40% of published cases, but common and recurrent facial features have not been reported [5,19].

The predominantly neurological phenotypic spectrum in BP1–BP2 carriers correlates with strong evidence that four genes included in the 15q11.2 region are variously implicated in axonal growth and neural connectivity function [3]. However, some issues may question the real impact of the 15q11.2 CNV on the phenotype in carriers. Large studies revealed that the BP1-BP2 CNV is not so rare and can be found in 0.5% to 1% of the general population [10]. Children affected by neurodevelopmental disorders inherited the CNV from a seemingly healthy parent approximately in 50% of cases and from a parent with nonspecific neuropsychiatric disorder in 35%. In less than 25% of cases, the CNV is de novo [20]. Unmasking of recessive mutations, unknown variants in other genomic regions, complex multigenic interactions, or parent-of-origin effects, are just some of the mechanisms proposed as an explanation for the wide phenotypic variability [21,22].

Our analysis did not reveal a significant correlation between the size of the 15q11.2 CNV and the severity of the patients’ symptoms. This finding may suggest that the observed clinical variability could be attributed to additional factors beyond the CNV size, potentially pointing to alternative diagnoses or underlying genetic conditions.

This study expands the investigation of genetic variants contributing to phenotype in a cohort of 15q11.2 CNV carriers with neurodevelopmental disorders. In three out of 35 (9%) children, CMA detected additional genomic arrangements, as similarly found in other cohorts [23]. In case 1 and case 2, the other CNV is likely the main cause of pathological features, considering the large size (4.16 Mb and 10.2 Mb, respectively) and the genomic region involved. In case 3, the coexistence of two susceptibility factors for neurodevelopmental disorders (15q11.2 duplication and 16p13.11 duplication) makes it difficult to attribute the impact of each CNV on the phenotype.

In the remaining 32 children, ES showed a very high detection rate (34%) of pathogenic or likely pathogenic variants (PV/LPV). In four additional cases (13%), a variant of uncertain significance has been reported (VOUS). The variants identified through ES provided insights into the specific clinical characteristics of each patient, as we filtered the reported variants to include only those aligning with the observed phenotypes in our cohort. This approach enhances our understanding of genotype-phenotype correlations, underscoring the importance of tailored genetic analysis in clinical settings. No mutation in one of the four genes (*NIPA1, NIPA2, CYFIP1, TUBGCP5*) involved in the CNV was found. We therefore excluded, at least in our cohort, the hypothesis that the 15q11.2 deletion or duplication unmasks recessive point mutations on the other allele [24]. Furthermore, ES did not reveal pathogenic variants in genes functionally interconnected or their paralogs, as proposed in a recent publication [21].

Most of the PV/LPV fell into genes involved in neurogenesis and associated with phenotypes that overlap the broad spectrum of clinical features typically attributed to the 15q11.2 CNV. We wondered if secondary findings were double diagnoses or alternative diagnoses in our patients. Genome-wide analyses are highlighting an increasing prevalence of patients whose phenotype is caused by two (or more) genetic conditions, with the frequency of double diagnoses now estimated at 2–7.5% [25]. However, in 34% of children in our cohort, we found a PV/LPV. This percentage is similar to the detection rate of ES in cohorts of patients affected by neurodevelopmental disorders without CNV [26]. These data allow us to conclude that the secondary findings are actually alternative diagnoses and that the previously detected 15q11.2 CNV was an obstacle to correct diagnostic framing.

The change of diagnosis has implications for both the clinical care of the child and for the reproductive risk of the family. While 15q.11.2 CNVs are typically inherited, with a 50% recurrence risk, ES identified de novo point mutations in 9 children (28%), lowering the risk to 1% for possible germinal mosaicism. In one child, compound heterozygous variants for a recessive condition were brought to light. In another child, an X-linked condition of maternal origin was identified.

However, ES did not point out alternative diagnoses in 21 out of 32 (66%) 15q11.2 CNV carriers. We investigated if some clinical features were a positive predictive factor for further PV/LPV, but we did not find any statistically significant correlations. Instead, we found a higher prevalence of autism spectrum disorder in patients without other CNVs in the CMA or variants in the ES analysis. The additional risk of the 15q11.2 CNV for ASD was previously estimated only at 0.3% for deletion and 0.8% for duplication [27,28]. This observation underscores the multifactorial nature of ASD, influenced by polygenic and environmental factors, thus complicating the identification of a singular pathogenic variant [29].

## 5. Conclusions

The ES analysis demonstrated a diagnostic yield of 34% in a cohort of pediatric patients with 15q11.2 CNV who are affected by neurodevelopmental disorders. This finding highlights the potential value of comprehensive genetic evaluation in this population. Although our study does not rule out the possibility that the BP1–BP2 CNV may play a role in the phenotype—possibly serving as a susceptibility factor—our results suggest that the secondary findings identified through ES should not be considered simply instances of double diagnosis. Instead, they likely represent the actual underlying causes of the observed pathological features in these patients.

Given the complexity of neurodevelopmental disorders, it is crucial to maintain an ongoing diagnostic process. We strongly recommend continuous clinical monitoring and further genetic analyses for all carriers of the 15q11.2 CNV who are experiencing neurodevelopmental challenges, whether these are isolated or accompanied by other clinical manifestations. The identification of alternative diagnoses can significantly influence management strategies and therapeutic approaches, as well as provide critical insights for family counseling regarding prognosis and recurrence risks.

In summary, our findings highlight the necessity for a tailored diagnostic framework that considers both the known genetic contributions and the potential for additional pathogenic variants. By adopting this comprehensive approach, we can enhance our understanding of the clinical variability in this cohort and improve the quality of care for affected individuals and their families.

## Data Availability

The data that support the findings of this study are available on request from the corresponding author. The data are not publicly available due to privacy or ethical restrictions.

## Supplementary Materials

The following supporting information can be downloaded at: https://www.mdpi.com/article/10.3390/genes15111441/s1, Table S1: Clinical, Instrumental, and Genetic Data of Patients.

## References

[1] Locke D.P., Segraves R., Nicholls R.D., Schwartz S., Pinkel D., Albertson D.G. (2004). BAC microarray analysis of 15q11-q13 rearrangements and the impact of segmental duplications. J. Med. Genet..

[2] Chai J.H., Locke D.P., Greally J.M., Knoll J.H.M., Ohta T., Dunai J., Yavor A., Eichler E.E., Nicholls R.D. (2003). Identification of four highly conserved genes between breakpoint hotspots BP1 and BP2 of the Prader-Willi/Angelman syndromes deletion region that have undergone evolutionary transposition mediated by flanking duplicons. Am. J. Hum. Genet..

[3] Rafi S.K., Butler M.G. (2020). The 15q11.2 BP1-BP2 Microdeletion (Burnside–Butler) Syndrome: In Silico Analyses of the Four Coding Genes Reveal Functional Associations with Neurodevelopmental Disorders. Int. J. Mol. Sci..

[4] Burnside R.D., Pasion R., Mikhail F.M., Carroll A.J., Robin N.H., Youngs E.L., Gadi I.K., Keitges E., Jaswaney V.L., Papenhausen P.R. (2011). Microdeletion/microduplication of proximal 15q11.2 between BP1 and BP2: A susceptibility region for neurological dysfunction including developmental and language delay. Hum. Genet..

[5] Cox D.M., Butler M.G. (2015). The 15q11.2 BP1-BP2 Microdeletion Syndrome: A Review. Int. J. Mol. Sci..

[6] Butler M.G. (2017). Clinical and genetic aspects of the 15q11.2 BP1–BP2 microdeletion disorder. J. Intellect. Disabil. Res..

[7] Rosenfeld J.A., Coe B.P., Eichler E.E., Cuckle H., Shaffer L.G. (2013). Estimates of penetrance for recurrent pathogenic copy-number variations. Genet. Med..

[8] Meossi C., Carrer A., Ciaccio C., Estienne M., Silipigni R., Sciacca F.L., Pantaleoni C., D’Arrigo S., Milani D. (2023). Clinical features and magnesium levels: Novel insights in 15q11.2 BP1–BP2 copy number variants. J. Intellect. Disabil. Res..

[9] Pezzani L., Marchetti D., Cereda A., Caffi L.G., Manara O., Mamoli D., Pezzoli L., Lincesso A.R., Perego L., Pellicioli I. (2018). Atypical presentation of pediatric BRAF RASopathy with acute encephalopathy. Am. J. Med. Genet. A.

[10] Richards S., Aziz N., Bale S., Bick D., Das S., Gastier-Foster J., Grody W.W., Hegde M., Lyon E., Spector E. (2015). Standards and guidelines for the interpretation of sequence variants: A joint consensus recommendation of the American College of Medical Genetics and Genomics and the Association for Molecular Pathology. Genet. Med..

[11] Murthy S., Nygren A., El Shakankiry H., Schouten J., Al Khayat A., Ridha A., Al Ali M. (2007). Detection of a novel familial deletion of four genes between BP1 and BP2 of the Prader-Willi/Angelman syndrome critical region by oligo-array CGH in a child with neurological disorder and speech impairment. Cytogenet. Genome Res..

[12] De Kovel C.G.F., Trucks H., Helbig I., Mefford H.C., Baker C., Leu C., Kluck C., Lie K., Hallmann K., Steffens K. (2010). Recurrent microdeletions at 15q11.2 and 16p13.11 predispose to idiopathic generalized epilepsies. Brain.

[13] Vanlerberghe C., Petit F., Malan V., Vincent-Delorme C., Bouquillon S., Boute O., Holder-Espinasse M., Delobel B., Duban B., Vallee L. (2015). 15q11.2 microdeletion (BP1–BP2) and developmental delay, behaviour issues, epilepsy and congenital heart disease: A series of 52 patients. Eur. J. Med Genet..

[14] van der Meer D., Sønderby I.E., Kaufmann T., Walters B., Abdellaoui A., Ames D., Amunts K., Andersson M., Armstrong N.J., Bernard M. (2020). Association of Copy Number Variation of the 15q11.2 BP1-BP2 Region With Cortical and Subcortical Morphology and Cognition Supplemental content. JAMA Psychiatry.

[15] Chu F.-C., Shaw S.W., Lee C.-H., Lo L.-M., Hsu J.-J., Hung T.-H. (2021). Adverse Perinatal and Early Life Outcomes following 15q11.2 CNV Diagnosis. Genes.

[16] Monteiro R.A., de Freitas M.L., Vianna G.S., de Oliveira V.T., Pietra R.X., Ferreira L.C., Rocha P.P., Gonçalves M.d.S., César G.d.C., Lima J.d.S. (2017). Major Contribution of Genomic Copy Number Variation in Syndromic Congenital Heart Disease: The Use of MLPA as the First Genetic Test. Mol. Syndr..

[17] Williams S.G., Nakev A., Guo H., Frain S., Tenin G., Liakhovitskaia A., Saha P., Priest J.R., Hentges K.E., Keavney B.D. (2020). Association of congenital cardiovascular malformation and neuropsychiatric phenotypes with 15q11.2 (BP1–BP2) deletion in the UK Biobank. Eur. J. Hum. Genet..

[18] Boen R., Kaufmann T., Frei O., van der Meer D., Djurovic S., Andreassen O.A., Selmer K.K., Alnæs D., Sønderby I.E. (2023). No signs of neurodegenerative effects in 15q11.2 BP1-BP2 copy number variant carriers in the UK Biobank. Transl. Psychiatry.

[19] Hashemi B., Bassett A., Chitayat D., Chong K., Feldman M., Flanagan J., Goobie S., Kawamura A., Lowther C., Prasad C. (2015). Deletion of 15q11.2(BP1-BP2) region: Further evidence for lack of phenotypic specificity in a pediatric population. Am. J. Med Genet. Part A.

[20] Cafferkey M., Ahn J.W., Flinter F., Ogilvie C. (2014). Phenotypic features in patients with 15q11.2(BP1-BP2) deletion: Further delineation of an emerging syndrome. Am. J. Med. Genet. A.

[21] Baldwin I., Shafer R.L., Hossain W.A., Gunewardena S., Veatch O.J., Mosconi M.W., Butler M.G. (2021). Genomic, Clinical, and Behavioral Characterization of 15q11.2 BP1-BP2 Deletion (Burnside-Butler) Syndrome in Five Families. Int. J. Mol. Sci..

[22] Davis K.W., Serrano M., Loddo S., Robinson C., Alesi V., Dallapiccola B., Novelli A., Butler M. (2019). Parent-of-Origin Effects in 15q11.2 BP1-BP2 Microdeletion (Burnside-Butler) Syndrome. Int. J. Mol. Sci..

[23] Maya I., Perlman S., Shohat M., Kahana S., Yacobson S., Tenne T., Agmon-Fishman I., Mater R.T., Salmon B.L., Halevy R.S. (2020). Should We Report 15q11.2 BP1-BP2 Deletions and Duplications in the Prenatal Setting?. J. Clin. Med..

[24] Maver A., Čuturilo G., Kovanda A., Miletić A., Peterlin B. (2019). Rare missense TUBGCP5 gene variant in a patient with primary microcephaly. Eur. J. Med. Genet..

[25] Rosina E., Pezzani L., Apuril E., Pezzoli L., Marchetti D., Bellini M., Lucca C., Meossi C., Massimello M., Mariani M. (2023). Comparison of first-tier whole-exome sequencing with a multi-step traditional approach for diagnosing paediatric outpatients: An Italian prospective study. Mol. Genet. Genom. Med..

[26] Srivastava S., Love-Nichols J.A., Dies K.A., Ledbetter D.H., Martin C.L., Chung W.K., Helen F., Thomas F., Lisa P., Han F. (2019). Meta-analysis and multidisciplinary consensus statement: Exome sequencing is a first-tier clinical diagnostic test for individuals with neurodevelopmental disorders. Genet. Med..

[27] Chaste P., Sanders S.J., Mohan K.N., Klei L., Song Y., Murtha M.T., Hus V., Lowe J.K., Willsey A.J., Moreno-De-Luca D. (2014). Modest Impact on Risk for Autism Spectrum Disorder of Rare Copy Number Variants at 15q11.2, Specifically Breakpoints 1 to 2. Autism Res..

[28] Genovese A., Butler M.G. (2023). The Autism Spectrum: Behavioral, Psychiatric and Genetic Associations. Genes.

[29] Kreiman B.L., Boles R.G. (2020). State of the Art of Genetic Testing for Patients With Autism: A Practical Guide for Clinicians. Semin. Pediatr. Neurol..

